# Comparative assessment of viral dynamic models for SARS‐CoV‐2 for pharmacodynamic assessment in early treatment trials

**DOI:** 10.1111/bcp.15518

**Published:** 2022-09-15

**Authors:** Akosua A. Agyeman, Tao You, Phylinda L. S. Chan, Dagan O. Lonsdale, Christoforos Hadjichrysanthou, Tabitha Mahungu, Emmanuel Q. Wey, David M. Lowe, Marc C. I. Lipman, Judy Breuer, Frank Kloprogge, Joseph F. Standing

**Affiliations:** ^1^ Infection, Immunity and Inflammation Research and Teaching Department, Great Ormond Street Institute of Child Health University College London London UK; ^2^ Beyond Consulting Ltd. Cheshire UK; ^3^ Medical Research Council UK; ^4^ Pfizer Sandwich Kent UK; ^5^ Department of Clinical Pharmacology St George's University of London London UK; ^6^ Department of Intensive Care St George's University Hospitals NHS Foundation Trust London UK; ^7^ Department of Infectious Disease Epidemiology, School of Public Health, Faculty of Medicine Imperial College London London UK; ^8^ Department of Infectious Diseases Royal Free Hospital London NHS Foundation Trust London UK; ^9^ Centre for Clinical Microbiology Division of Infection and Immunity University College London London UK; ^10^ Department of Clinical Immunology Royal Free London NHS Foundation Trust London UK; ^11^ Institute of Immunity and Transplantation University College London, Royal Free Campus London UK; ^12^ Department of Respiratory Medicine Royal Free London NHS Foundation Trust London UK; ^13^ UCL Respiratory University College London, Royal Free Campus London UK; ^14^ Department of Microbiology Great Ormond Street Hospital for Children London UK; ^15^ Institute for Global Health University College London London UK; ^16^ Department of Pharmacy Great Ormond Street Hospital for Children London UK

**Keywords:** COVID‐19, model performance, pharmacometrics, SARS‐COV‐2, viral dynamics

## Abstract

Pharmacometric analyses of time series viral load data may detect drug effects with greater power than approaches using single time points. Because SARS‐CoV‐2 viral load rapidly rises and then falls, viral dynamic models have been used. We compared different modelling approaches when analysing Phase II‐type viral dynamic data. Using two SARS‐CoV‐2 datasets of viral load starting within 7 days of symptoms, we fitted the slope‐intercept exponential decay (SI), reduced target cell limited (rTCL), target cell limited (TCL) and TCL with eclipse phase (TCLE) models using nlmixr. Model performance was assessed via Bayesian information criterion (BIC), visual predictive checks (VPCs), goodness‐of‐fit plots, and parameter precision. The most complex (TCLE) model had the highest BIC for both datasets. The estimated viral decline rate was similar for all models except the TCL model for dataset A with a higher rate (median [range] day^−1^: dataset A; 0.63 [0.56–1.84]; dataset B: 0.81 [0.74–0.85]). Our findings suggest simple models should be considered during pharmacodynamic model development.

**What is already known about this subject**

- The target cell limited (TCL) model has been widely used to support antiviral development for respiratory infections.
- For SARS‐CoV‐2, extensions and simplifications of the T model have been reported in recent studies but model selection or justification of the chosen pharmacodynamic model is often lacking.

**What this study adds**

- This study compared the simplified and extended forms of the TCL model and found no advantage of the more complex (TCL, TCLE) models over simplified forms (SI, rTCL), which could inform the selection of a suitable modelling approach for SARS‐CoV‐2 viral dynamics.

## 1 INTRODUCTION

The COVID‐19 pandemic continues to threaten public health largely now due to new variants of concern with increasing ability to evade antibody responses. Most importantly, these variants challenge vaccination efforts to halt the pandemic, thereby necessitating efforts to develop new antivirals as well as repurposing of existing antiviral therapies.^1^

So far, the ongoing development of novel antivirals is promising, albeit drug development processes are time‐consuming. Drug repurposing is a time‐saving approach as clinical efficacy and safety data are already known for other therapeutic indications.^2^ Nonetheless, the push for repurposing therapies for SAR‐CoV‐2 has been hampered by clinical inefficiencies such as non‐randomised placebo‐controlled trials and an overemphasis on hospitalised patients.^2^, ^3^

As with other respiratory viral infections, an understanding of SARS‐CoV‐2 viral dynamics could shape the future of potential treatment options to identify antivirals which can disrupt viral replication. The target cell limited (TCL) model has previously been used to support antiviral development for respiratory infections.^4^, ^5^ For SARS‐CoV‐2, extensions and simplifications of the TC model have been described in recent studies.^6^, ^7^, ^8^, ^9^, ^10^

During pharmacokinetic model building, common practice involves starting with the simplest model (often one‐compartment) and then adding complexity (further compartments) where data supports this. The goal is to find a model that adequately describes the data and from which important secondary parameters such as area under the curve (AUC) or highest observed concentration (*C*_max_) can be derived. SARS‐CoV‐2 viral pharmacodynamic modelling has so far often not taken this approach, in that only one model is often considered. As Phase II type trials of repurposed and novel antivirals read out, it is important to consider a model‐building approach that is sufficient to characterise viral decline rate as the clinical endpoint of interest. And for most pharmacodynamic models, characterisation of the infected cells death rate (i.e., *δ*) is the main driver of viral decline rate since often virus clearance (e.g., *c* in the TCL model) is much faster than viral production rate.^7^, ^8^, ^9^, ^10^

Therefore, we aimed to compare the performance of different published viral dynamic models for SARS‐CoV‐2 in predicting the rate of viral decline to inform the model selection for pharmacodynamic model development of Phase II trial of antiviral treatment options.

## 2 METHODS

### 2.1 Data

Two published datasets on patients with COVID‐19 were obtained from a recent systematic review (Gastine et al., herein referred to as dataset A)^6^ and a prospective cohort study (Néant et al., herein referred to as dataset B).^7^ Details of the patients' characteristics have been published previously.^6^, ^7^ Briefly, for dataset A, a mild disease state was presented by the majority of patients with one reported death. Viral load samples were obtained from either upper or lower respiratory tract, blood, stool, urine, ocular, and breast milk. Patients were either untreated or received treatment including antiviral, antibiotic, hydroxychloroquine, and interferon. For dataset B, patients were hospitalised in either conventional or intensive care units and a total of 78 deaths were reported during follow‐up. Nasopharyngeal viral load samples were utilised and patients were on treatment including antiviral, antibiotic, antifungal or corticosteroid. The extracted viral load data were limited to 14 days post‐symptom onset to replicate the time window of a 7‐day treatment course started a maximum of 7 days after symptom onset. Additionally, for dataset A, viral load data were limited to upper respiratory sampling sites and untreated patients.

### 2.2 Viral dynamic models for SARS‐CoV‐2

**FIGURE 1:**
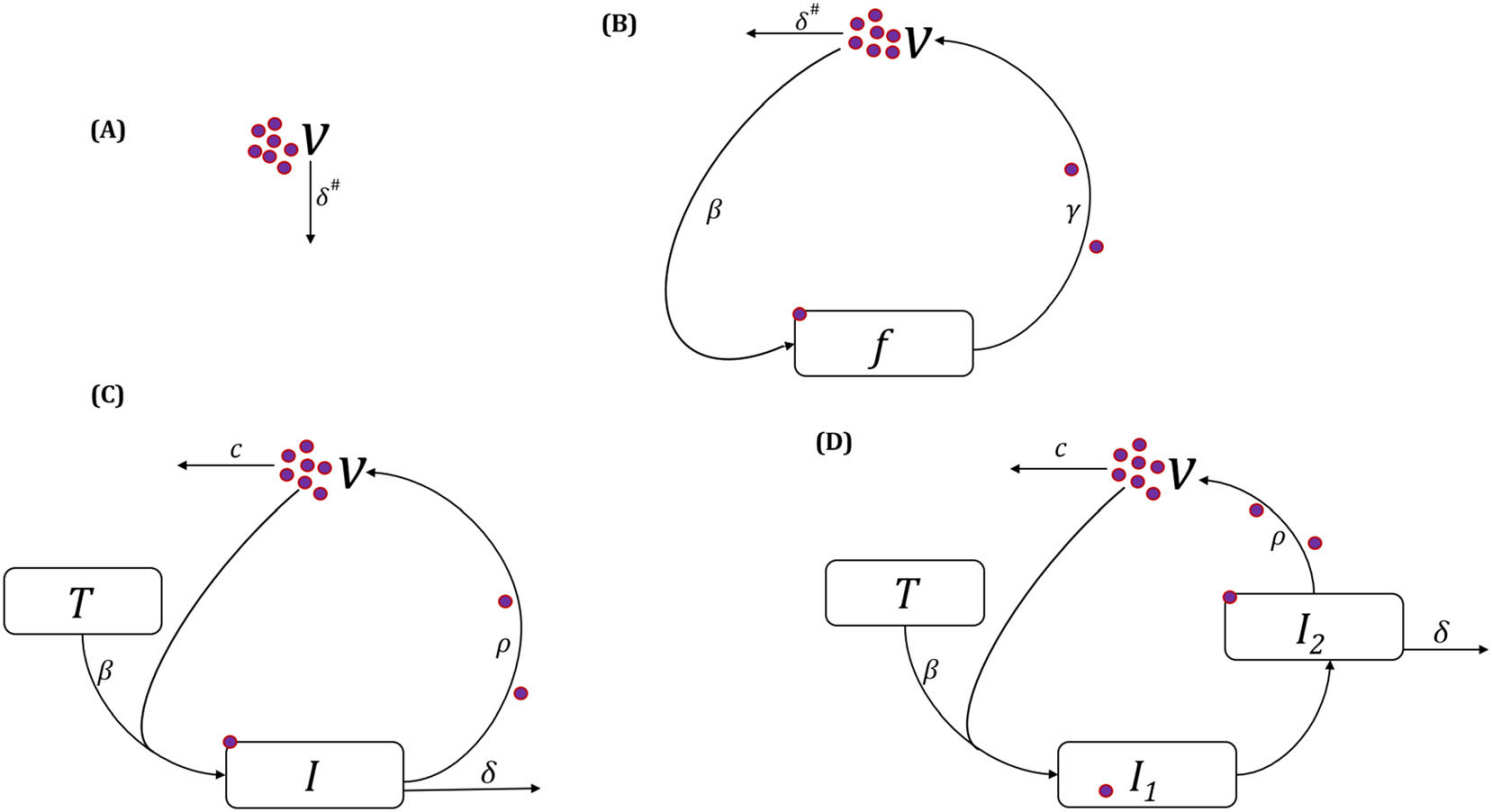
(A) Slope‐intercept exponential decay model: Viral particles (*V*) are eliminated by an overall viral elimination rate of *δ*. (B) Reduced target cell limited model: Fraction of target cells remaining (*f*) are infected by viral particles at a rate of *β* to release viruses at a maximum rate constant of *γ* and cleared at an overall viral elimination rate of *δ*. (C) Target cell limited model: Uninfected target cells (*T*) are infected by viral particles at an infection rate of *β* and become productively infected cells (*I*) and release viruses at a rate of *ρ* with a viral clearance rate of *c*. Productively infected cells die at a rate of *δ*. (D) Target cell limited model with eclipse phase: Uninfected target cells (*T*) are infected by viral particles at an infection rate of *β* and become latently infected cells during an incubation period (*I*_1_) and convert to productively infected cells (*I*_2_) at a rate of *k*. *I*_2_ subsequently release viruses at a rate of *ρ* with a viral clearance rate of *c*. Productively infected cells die at a rate of *δ*. ^#^For models (A) and (B), the assumption of quasi‐steady state between *I* and *V* due to the typically faster *c* than *δ* translates *δ* as the overall viral elimination rate as previously described.^6^, ^8^

Schematic diagrams of the slope‐intercept exponential decay (SI), reduced target cell limited (rTCL), target cell limited (TCL), and TCL with eclipse phase (TCLE) models are shown in Figure 1. Details of the mathematical expressions underlying all four models which are characterised by two (SI), five (rTCL), seven (TCL) and nine (TCLE) parameters are expressed in Equations (1), (2), (3) and (4), respectively.^4^, ^6^, ^7^, ^8^, ^9^, ^10^ *T*, *I*, *I*_1_, *I*_2_, *V* and *f* are uninfected target cells, infected target cells, latently infected cells, productively infected cells, viral particles, and fraction of target cells remaining, respectively. The parameters *β*, *δ*, *ρ*, *c*, *γ* and *k* represent the rate constant for virus infection, death rate of infected cells, viral production rate, clearance rate of viral particles, maximum viral replication rate, and conversion rate from *I*_1_ to *I*_2_, respectively. For the SI and rTCL models, the assumption of quasi‐steady state between *I* and *V* due to the typically faster *c* than *δ* translates *δ* as the overall viral elimination rate as previously described.^6^, ^8^

(1) $$\frac{\mathrm{dV}\left(t\right)}{\mathrm{dt}}=-\mathrm{\delta V}\left(t\right).$$

(2) $$\begin{array}{c} \frac{\mathrm{df}\left(t\right)}{\mathrm{dt}}=-\mathrm{\beta f}\left(t\right)V\left(t\right).\\ \frac{\mathrm{dV}\left(t\right)}{\mathrm{dt}}=\mathrm{\gamma f}\left(t\right)V\left(t\right)-\mathrm{\delta V}\left(t\right).\;\text{where}\;\gamma =\mathrm{\rho \beta T}\left(0\right)/c. \end{array}$$

(3) $$\begin{array}{c} \frac{\mathrm{dT}\left(t\right)}{\mathrm{dt}}=-\mathrm{\beta T}\left(t\right)V\left(t\right).\\ \frac{\mathrm{dI}\left(t\right)}{\mathrm{dt}}=-\mathrm{\beta T}\left(t\right)V\left(t\right)-\mathrm{\delta I}\left(t\right).\\ \frac{\mathrm{dV}\left(t\right)}{\mathrm{dt}}=\mathrm{\rho I}\left(t\right)-\mathrm{cV}\left(t\right). \end{array}$$

(4) $$\begin{array}{c} \frac{\mathrm{dT}\left(t\right)}{\mathrm{dt}}=-\mathrm{\beta T}\left(t\right)V\left(t\right).\;\\ \begin{array}{c} \frac{dI_{1}\left(t\right)}{\mathrm{dt}}=-\mathrm{\beta T}\left(t\right)V\left(t\right)-kI_{1}\left(t\right).\\ \frac{dI_{2}\left(t\right)}{\mathrm{dt}}=kI_{1}\left(t\right)-\delta I_{2}\left(t\right). \end{array}\\ \frac{\mathrm{dV}\left(t\right)}{\mathrm{dt}}=\rho I_{2}\left(t\right)-\mathrm{cV}\left(t\right)-\mathrm{\beta T}\left(t\right)V\left(t\right). \end{array}$$

Initial estimates for model parameters were derived from published studies from which model structures were utilised for evaluation.^4^, ^6^, ^7^, ^8^, ^9^, ^10^ Time since symptom onset as reported by the authors of the included datasets was utilised for model fitting. For TCL and TCLE models, since time of infection is unidentifiable, the incubation period (i.e., time from infection to onset of symptoms) employed was based on sensitivity analysis of incubation periods ranging from 0.5 to 14 days.

The basic reproduction number (*R*_0_) was calculated based on the estimated model parameters and expressed in Equations (5) (SI and rTCL models),^8^ (6) (TCL model)^5^ and (7) (TCLE model).^10^ The duration of virus production (*L*) was also derived for all models using Equation (8).^10^

(5) $$R_{0}=\frac{\gamma }{\delta }.$$

(6) $$R_{0}=\frac{\rho \beta T_{0}}{\mathrm{c\delta}}.$$

(7) $$R_{0}=\frac{\rho \beta T_{0}}{\delta \left(c+\beta T_{0}\right)}.$$

(8) $$L=\frac{1}{\delta }.$$

### 2.3 Model‐fitting assessment

Each non‐linear mixed‐effects model was fitted to viral load data using the stochastic approximation expectation maximisation in R package nlmixr (version 2.0.6). For each subject *i*, the parameter value is θ_*i*_ (= θ × *e* ^*Πi*^), where θ and *e* ^*Πi*^ are fixed and random effects respectively. The inclusion of fixed and random effects accounts for interindividual variability which follows a log‐normal distribution. Random effect terms were specified for each estimated parameter using the full covariance matrix structure where possible. Otherwise, when convergence was not achieved, the variance was specified individually for the parameters without estimating correlations. Viral loads were log transformed with residual error assumed to follow a normal distribution. Viral loads below the limit of detection (LOD) were censored between a predefined “LIMIT” (i.e., log [0.001] copies/mL) and the LOD as per the censoring method described for nlmixr.^11^ Model performance was assessed via Bayesian information criterion (BIC), visual predictive checks (VPCs), goodness‐of‐fit plots, and parameter precision. A lower comparative BIC value indicated a better model fit. For the generation of VPCs, we chose the most frequently reported LOD value for each dataset as it is not possible to make VPCs with multiple LOD values. However, the LOD values as reported in the included studies were used in the models.

## 3 RESULTS

In all, 252 patients with 747 viral load samples from dataset A and 321 patients with 563 viral load samples from dataset B were extracted. The TCL model achieved the lowest BIC value (4155 *vs* 4185 [SI model], 4254 [rTCL model] and 4383 [TCLE model]) for dataset A. For dataset B, the SI modelyielded the lowest BIC value (3432 *vs* 3546 [rTCL model], 3551 [TCL model] and 3665 [TCLE model]) (Table S1 in the Supporting Information). Based on sensitivity analysis, an incubation period of 0.5 days yielded the lowest BIC for TCL and TCLE models with dataset A. Likewise, with dataset B, the lowest BIC was achieved with incubation periods of 1 day and 1.5 days for TCL and TCLE models, respectively (Figures S1 and S2 in the Supporting Information).

Both datasets recorded parameter estimates with adequate precision for all models except TCL, which yielded the highest imprecision for *c* (%RSE; 130%) with dataset B (Tables S2 and S3 in the Supporting Information). All models predicted similar *δ* values except the TCL model for dataset A with a higher rate. For dataset A, *δ* was in the range 0.56–1.84 day^−1^ (median: 0.63 day^−1^) and that of dataset B was 0.74–0.85 (median: 0.81 day^−1^). *R*_0_ for the TCLE model indicated very high within‐host reproduction numbers of 1995 (dataset A) and 2908 (dataset B). Similarly, the *R*_0_ for TCL model was 16 787 for dataset A and 64 894 for dataset B, indicating parameter estimates that were not physiologically plausible. The high *R*_0_ for the TCLE model were considerably lower (22.21 for dataset A and 24.09 for dataset B) when *ρ* was fixed to 10 copies/mL.day^−1^. In contrast, the *R*_0_ for TCL model remained high following evaluation with fixed parameters. For the rTCL model, low *R*_0_ values of 1.79 (dataset A) and 1.23 (dataset B) were estimated. *L* ranged from 0.59 to 1.85 days across all models for both datasets (Tables S2 and S3 in the Supporting Information).

All four models yielded goodness‐of‐fit plots that were in satisfactory agreement with trends observed with both datasets (Figure S3 in the Supporting Information). VPC plots were adequate for all models for dataset B. However, VPC plots for the TCL and TCLE models displayed poor predictive performance on the 5th percentile below the limit of detection (LOD) at early time points for dataset A (Figure S4 in the Supporting Information).

## 4 DISCUSSION

In the present study, the model performance of the TCL model including both extended and simplified forms was evaluated with two datasets from patients infected with COVID‐19. Overall, based on the datasets employed here, our results showed no advantage of the more complex (TCL, TCLE) models over the simplified forms (SI, rTCL) for the characterisation of SARS‐CoV‐2 viral dynamics in estimating the death rate of infected cells. This observation may be attributed to the parsimony and identifiability of the different model structures.

The complexity of viral dynamics may suggest more complex models to include all biologically plausible effects. However, the proposal of such models may result in overparameterised models with identifiability problems. For example, a ten‐equation model with 27 parameters has previously been reported for influenza A infection.^12^ Indeed, to ensure identifiability of all the parameters would require almost all variables (i.e., viral load per epithelial cell, proportion of healthy cells, proportion of infected cells, activated antigen presenting cells per homeostatic level, interferons per homeostatic level of macrophages, proportion of resistant cells, effector cells per homeostatic level, plasma cells per homeostatic level, antibodies per homeostatic level and antigenic distance) to be quantified which may not be practically and ethically feasible.

Likewise, for SARS‐CoV‐2, having more complex models may be useful for hypothesis testing but particularly challenging for fitting data where strong prior information on required parameters may be lacking. Thus, in the proposed rTCL model to characterise SARS‐CoV‐2 viral dynamics, Kim et al.,^8^ indicate that such reduced structure may not necessitate the inclusion of further compartments to describe immune effects as the structure implicitly captures innate responses that are expressed via model parameters such as infection rate. Also, Hernandez‐Vargas and Velasco‐Hernandez^13^ have reported a minimalist two‐compartment model for SARS‐CoV‐2 and its immune response, which had lower Akaike information criterion (AIC) values compared to TCL model.

Regarding model structure identifiability, time of infection is unidentifiable for TCL and TCLE models, and therefore incubation period is fixed based on sensitivity analysis or estimates from epidemiological studies. However, fixing the time of infection may not always resolve identifiability problems. In this study, although the incubation period was fixed to 0.5 days (TCLE model, dataset A) and 1 day (TCL model, dataset B) based on low BIC values, this timeframe was still unidentifiable as other days had similar BIC values. Fixing the incubation period using epidemiological estimates may also be biased by the uncertainty of exposure time based on recall.^14^ Thus, following structural identifiability analysis, Gastine et al.^6^ opted for rTCL model over TCL model for SARS‐CoV‐2, stating that the TCL model is structurally unidentifiable except *T*, *β* or *ρ* initial conditions are known.

Furthermore, the different incubation periods observed in the two datasets may suggest that viral dynamics and incubation period may vary with patient characteristics. A global meta‐analysis involving 53 studies by Cheng et al.^15^ indicated that incubation period varied across different patient age groups with a shorter incubation period among middle‐aged individuals (41–60 years). Also, the UK human challenge study in younger adults (18–29 years) reported a shorter incubation period of <2 days.^16^ Despite the limited generalisability of the human challenge study, their finding on the incubation period is consistent with the shorter incubation period (0.5–1.5 days) observed with the TCL and TCLE models for datasets A and B. Hence, the impact of patient characteristics on SARS‐CoV‐2 incubation period requires further exploration.

The estimates for *δ* across the different models for both datasets were also largely consistent with those previously reported for SARS‐CoV‐2 (range: 0.27–2.29 day^−1^).^7^, ^8^, ^14^ Of note, an alternative approach known as model averaging has been described for viral dynamic models where different models yielding similarly good fits are simultaneously utilised to account for model uncertainty.^17^ Although this approach may be reasonable, such complexity may not be required as the primary focus of viral dynamic models is the estimation of *δ*, which can equally be well characterised by simpler models, as seen here.

There are some limitations worth noting in this study. Firstly, only two datasets were evaluated and therefore our results may not be universally representative. Secondly, *R*_0_ was poorly estimated with the datasets employed in this study and as such the results should be interpreted with caution. Thirdly, participants in the two datasets were recruited prior to the emergence of SARS‐CoV‐2 variants of concern and, therefore, the results here ought to be interpreted within this context. Further studies should explore the performance of these models with SARS‐CoV‐2 emerging variants. In addition, our analysis was restricted to models proposed to describe antiviral effects in clinical trials and we did not test viral dynamic models from epidemiological studies,^18^, ^19^ which would be interesting to address in future work. Future studies may also consider a joint pharmacometric and epidemiological modelling approach to broaden the understanding of SARS‐CoV‐2 viral dynamics. Finally, we did not compare the performance of the different models in addressing other potential goals in viral dynamics modelling such as detecting antiviral effects and the impact of timing of therapeutic interventions on treatment outcomes. Such evaluations may therefore necessitate the use of more complex models and a minimalist model may not be the best choice. In such context, complex models may be considered, particularly where their structural identifiability could be improved without compromising the intended modelling goal.

In conclusion, as shown in the present study, we found no advantage of the complex models over simplified forms. This emphasises the need to explore both simplified and extended models to ascertain the most appropriate pharmacodynamic model development for SARS‐CoV‐2 viral dynamics.

## COMPETING INTERESTS

The authors declare there are no conflicts of interest.

## CONTRIBUTORS

A.A. drafted the original writing of the manuscript. A.A. and J.S. were involved in data preparation, data checking and data analysis. All authors were involved in the study design and interpretation of results and preparation of the final manuscript.

## Supporting information


**Table S1:** Quality of model fit evaluated by the Bayesian information criterion (BIC)
**Table S2:** Parameter estimates for non‐linear mixed effect model fitted to viral load for Gastine et al. (dataset A).
**Table S3:** Parameter estimates for non‐linear mixed effect model fitted to viral load for Néant et al. (dataset B).
**Figure S1:** Sensitivity analysis for evaluating the incubation period for the target cell limited (TCL) and TCL with eclipse phase (TCLE) models with Gastine et al. (dataset A). BIC, Bayesian information criterion.
**Figure S2:** Sensitivity analysis for evaluating the incubation period for the target cell limited (TCL) and TCL with eclipse phase (TCLE) models with Néant et al. (dataset B). BIC, Bayesian information criterion.
**Figure S3:** Goodness‐of‐fit plots for non‐linear mixed effect model fitted to viral load. Grey dots are observed viral loads. Black dots are observed viral loads below the limit of detection (LOD). The black line indicates the line of identity. The red line represents the smooth curve. Panel (A) represents data extracted from Gastine et al. (dataset A). Panel (B) represents data extracted from Néant et al. (dataset B). SI: slope‐intercept exponential decay model; rTCL: reduced target cell limited model; TCL: target cell limited model; TCLE: TCL with eclipse phase model. IPRED: individual predicted viral load; PRED: population predicted viral load; NPDE: normalised prediction distribution errors; DV: observed viral loads; ln: natural logarithm.
**Figure S4:** Visual predictive checks for non‐linear mixed effect model fitted to viral load. Circles are observed viral loads. The red line indicates the limit of detection (LOD). In the top plot, the black line is the 50th percentile of the observed data. The broken black lines represent the 95th and 5th percentiles of the observed data. The shaded areas represent the 90% prediction interval of the 95th, 50th and 5th percentiles of the simulated data. In the bottom plot, the black line represents the observed proportion of viral loads below the LOD, and the shaded area represents the 90% prediction interval of the simulated proportion of viral loads below the LOD. Panel (A) represents data extracted from Gastine et al. (dataset A). Panel (B) represents data extracted from Néant et al. (dataset B). SI: slope‐intercept exponential decay model; rTCL: reduced target cell limited model; TCL: target cell limited model; TCLE: TCL with eclipse phase model; ln: natural logarithm.

## Data Availability

The underlying data and analysis code are available at: https://github.com/ucl-pharmacometrics/SARS-CoV-2-viral-dynamic-models-comparison.
