# Supplementary material for: Comparative assessment of viral dynamic models for SARS‐CoV‐2 for pharmacodynamic assessment in early treatment trials
Source: Br J Clin Pharmacol. 2022 Sep 15:10.1111/bcp.15518. Online ahead of print. doi: 10.1111/bcp.15518 (PMC9538685; doi:10.1111/bcp.15518)
Supplement: Supplementary file 1 — Table S1: Quality of model fit evaluated by the Bayesian information criterion (BIC) Table S2: Parameter estimates for non‐linear mixed effect model fitted to viral load for Gastine et al. (dataset A). Table S3: Parameter estimates for non‐linear mixed effect model fitted to viral load for Néant et al. (dataset B). Figure S1: Sensitivity analysis for evaluating the incubation period for the target cell limited (TCL) and TCL with eclipse phase (TCLE) models with Gastine et al. (dataset A). BIC, Bayesian information criterion. Figure S2: Sensitivity analysis for evaluating the incubation period for the target cell limited (TCL) and TCL with eclipse phase (TCLE) models with Néant et al. (dataset B). BIC, Bayesian information criterion. Figure S3: Goodness‐of‐fit plots for non‐linear mixed effect model fitted to viral load. Grey dots are observed viral loads. Black dots are observed viral loads below the limit of detection (LOD). The black line indicates the line of identity. The red line represents the smooth curve. Panel (A) represents data extracted from Gastine et al. (dataset A). Panel (B) represents data extracted from Néant et al. (dataset B). SI: slope‐intercept exponential decay model; rTCL: reduced target cell limited model; TCL: target cell limited model; TCLE: TCL with eclipse phase model. IPRED: individual predicted viral load; PRED: population predicted viral load; NPDE: normalised prediction distribution errors; DV: observed viral loads; ln: natural logarithm. Figure S4: Visual predictive checks for non‐linear mixed effect model fitted to viral load. Circles are observed viral loads. The red line indicates the limit of detection (LOD). In the top plot, the black line is the 50th percentile of the observed data. The broken black lines represent the 95th and 5th percentiles of the observed data. The shaded areas represent the 90% prediction interval of the 95th, 50th and 5th percentiles of the simulated data. In the bottom plot, the black line represents th [file BCP-9999-0-s001.docx]

**Supplementary materials**

| **Model** | **BIC** | |
| --- | --- | --- |
|  | **Gastine et al (dataset A)** | **Neant et al (dataset B)** |
| SI | 4185 | 3432 |
| rTCL | 4254 | 3546 |
| TCL | 4155 | 3551 |
| TCLE | 4383 | 3665 |

**Table S1:** Quality of model fit evaluated by the Bayesian information criterion (BIC)

SI: slope-intercept exponential decay model; rTCL: reduced target cell limited model; TCL: target cell limited model; TCLE: TCL with eclipse phase.

| **Model Parameters** | | **Estimate (%RSE)** | | | |
| --- | --- | --- | --- | --- | --- |
| **Parameter name** | **Symbol (unit)** | **SI** | **rTCL** | **TCL** | **TCLE** |
| Viral load at symptom onset | *V*(0) (copies/ml) | 1.8 ×10^7^ (2.3) | 1.46 ×10^7^ (2.4) | - | - |
| Rate constant for virus infection | *β* ((copies/ml) ^−1^.day^−1^ ) | - | 5.0 ×10^-4^ (22.3) | 7.06 ×10^-4^ (15.6) | 7.76 ×10^-5^ (6.2)  2.28 ×10^-4^ (6.4)* |
| Death rate of infected cells | *δ* (day^−1^ )^#^ | 0.56 (13.6) | 0.56 (12.6) | 1.84 (50.3) | 0.69 (12.6)  0.43 (14.7)* |
| Viral production rate | *ρ* (copies/ml.day^−1^) | - | - | 0.28 (35.7) | 2.96 ×10^3^ (4.3) |
| Viral clearance rate | *c* (day^−1^ ) | - | - | 0.64 (13.8) | 11.6 (9.4)  1.4 (54.4)* |
| Maximum rate constant for viral replication | *γ* (day^−1^ ) | - | 1 (fixed) | - | - |
| Reproductivity rate of infected cells | *k* (day^−1^ ) | - | - | **-** | 9.23 (10.6)  50.8 (13.6)* |
| Basic reproduction number | *R*_0_ | - | 1.79 | 16787 | 1995  22.21* |
| Duration of virus production | *L* (day (s)) | 1.79 | 1.79 | 0.54 | 1.45  2.33* |

**Table S2:** Parameter estimates for non-linear mixed effect model fitted to viral load for Gastine et al (dataset A).

SI: slope-intercept exponential decay model; rTCL: reduced target cell limited model; TCL: target cell limited model; TCLE: TCL with eclipse phase. Initial conditions at infection: *f*(0) = 1 (for rTCL) (1); *T*(0) = 1× 10^8^ cells/ml, *I*(0) = 0 cells/ml, *V*(0) = 1 copies/ml (for TCL) (2); *T*(0) = 1.3 × 10^5^ cells/ml, *I_1_*(0) = 1/30 cells/ml, *I_2_*(0) = 0 cells/ml, *V*(0) = 0.1 copies/ml (for TCLE) (3,4). *Values obtained after fixing *ρ* to 10 copies/ml.day^-1^. ^#^*δ* translates as the overall viral elimination rate for SI and rTCL models (5). -, not applicable.

**Table S3:** Parameter estimates for non-linear mixed effect model fitted to viral load for Néant et al (dataset B).

| **Model Parameters** | | **Estimate (%RSE)** | | | |
| --- | --- | --- | --- | --- | --- |
| **Parameter name** | **Symbol (unit)** | **SI** | **rTCL** | **TCL** | **TCLE** |
| Viral load at symptom onset | *V*(0) (copies/ml) | 1.89 ×10^8^ (2.4) | 1.3 ×10^8^ (2) | - | - |
| Rate constant for virus infection | *β* ((copies/ml) ^−1^.day^−1^ ) | - | 1.27 ×10^-3^ (14.2) | 1.54 ×10^-3^ (17.1) | 9.79 ×10^-5^ (7.8)  2.08 ×10^-4^ (16.4)* |
| Death rate of infected cells | *δ* (day^−1^ )^#^ | 0.85 (32.5) | 0.80 (16.1) | 0.74 (25.3) | 0.81 (12.4)  0.34 (17.9)* |
| Viral production rate | *ρ* (copies/ml.day^−1^) | - | - | 0.29 (21.4) | 2.84 ×10^3^ (3.5) |
| Viral clearance rate | *c* (day^−1^ ) | - | - | 0.93 (130) | 2.62 (10)  2.73 (40.7)* |
| Maximum rate constant for viral replication | *γ* (day^−1^ ) | - | 1 (fixed) | - | - |
| Reproductivity rate of infected cells | *k* (day^−1^ ) | - | - | **-** | 8.87 (9.9)  16.7 (39.2)* |
| Basic reproduction number | *R*_0_ | - | 1.25 | 64894 | 2908  24.09* |
| Duration of virus production | *L* (day (s)) | 1.18 | 1.25 | 1.35 | 1.23  2.44* |

SI: slope-intercept exponential decay model; rTCL: reduced target cell limited model; TCL: target cell limited model; TCLE: TCL with eclipse phase. Initial conditions at infection: *f*(0) = 1 (for rTCL) (1); *T*(0) = 1× 10^8^ cells/ml, *I*(0) = 0 cells/ml, *V*(0) = 1 copies/ml (for TCL) (2); *T*(0) = 1.3 × 10^5^ cells/ml, *I_1_*(0) = 1/30 cells/ml, *I_2_*(0) = 0 cells/ml, *V*(0) = 0.1 copies/ml (for TCLE) (3,4). *Values obtained after fixing *ρ* to 10 copies/ml.day^-1^. ^#^*δ* translates as the overall viral elimination rate for SI and rTCL models (5). -, not applicable.


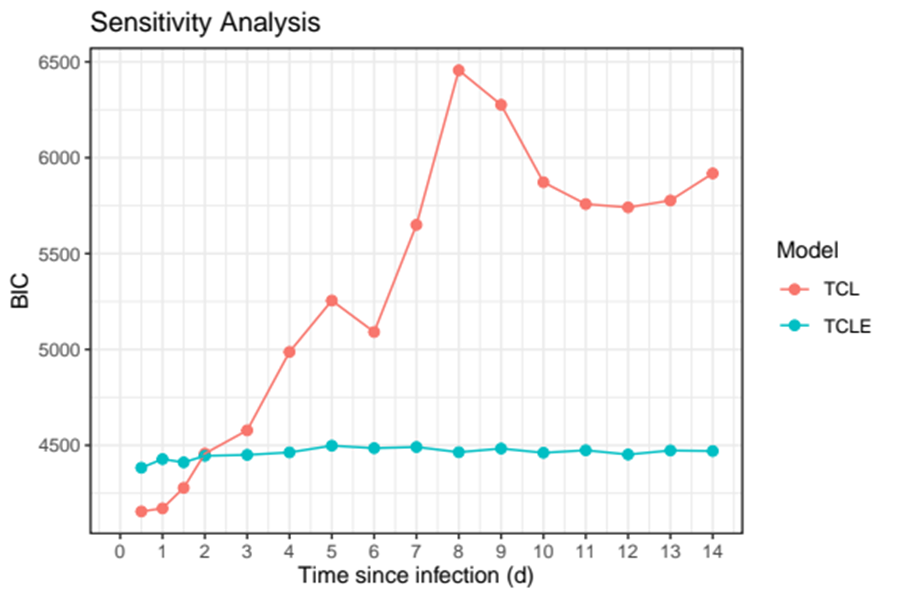


**Figure S1:** Sensitivity analysis for evaluating the incubation period for the target cell limited (TCL) and TCL with eclipse phase (TCLE) models with Gastine et al (dataset A). BIC, Bayesian information criterion.


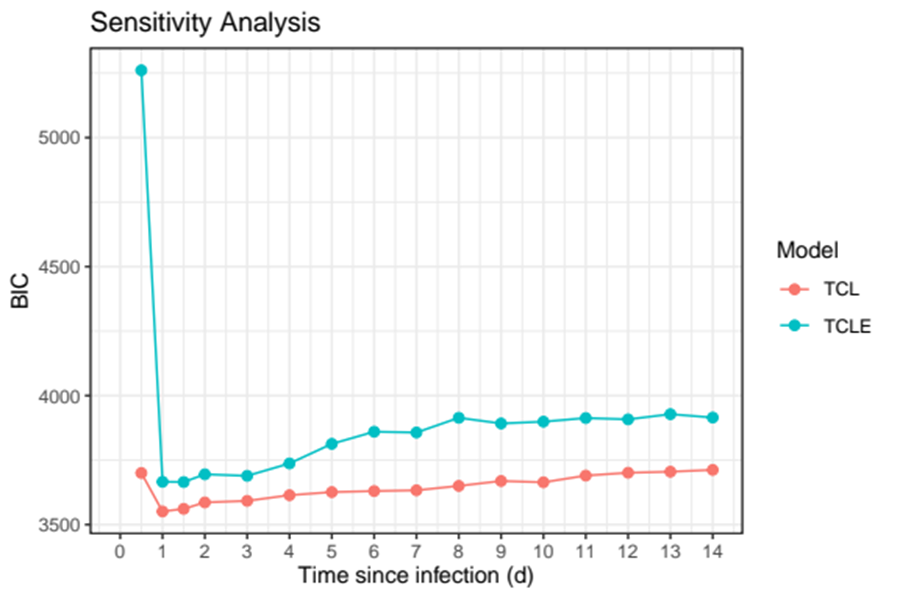


**Figure S2:** Sensitivity analysis for evaluating the incubation period for the target cell limited (TCL) and TCL with eclipse phase (TCLE) models with Néant et al (dataset B). BIC, Bayesian information criterion.


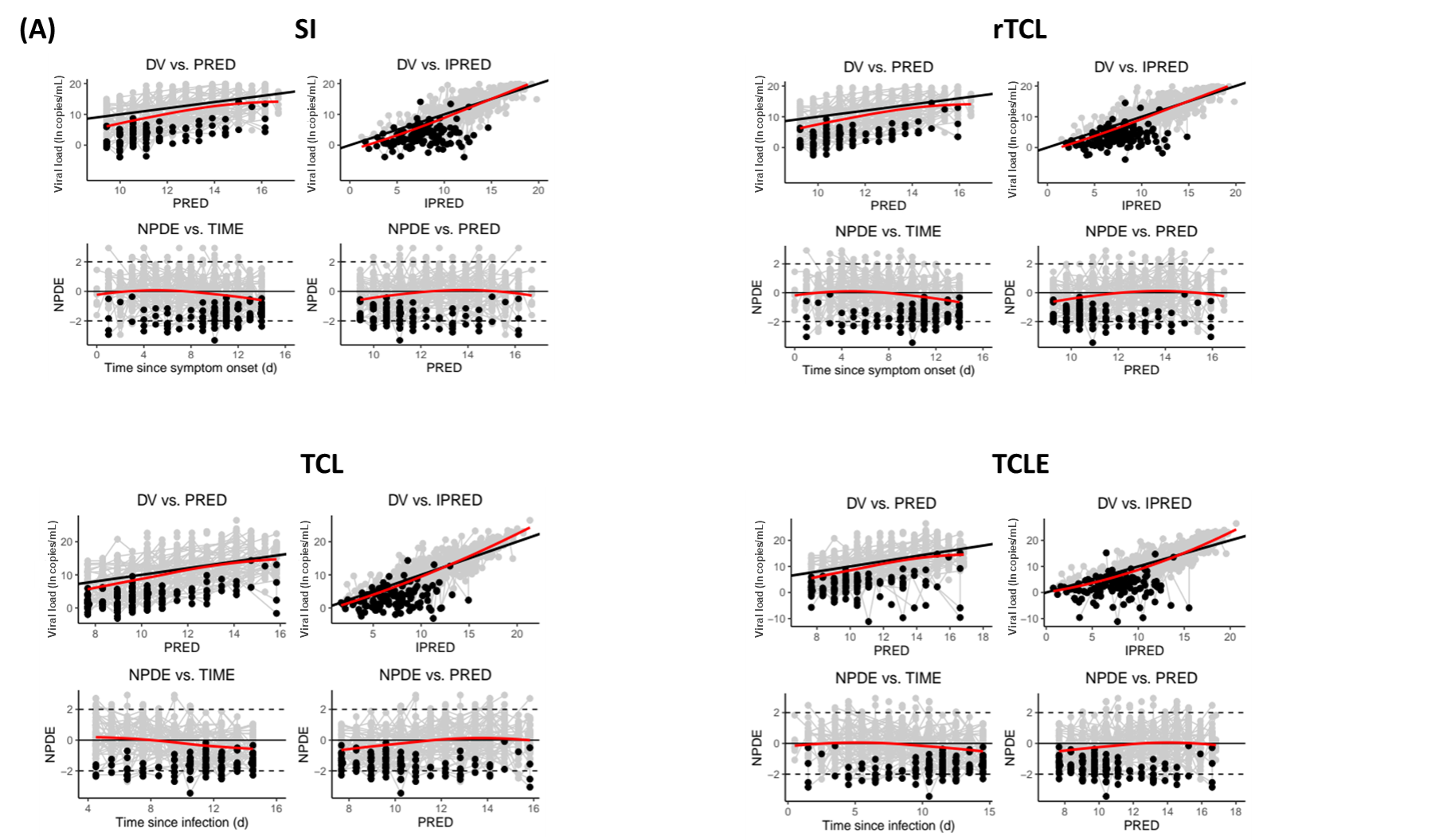


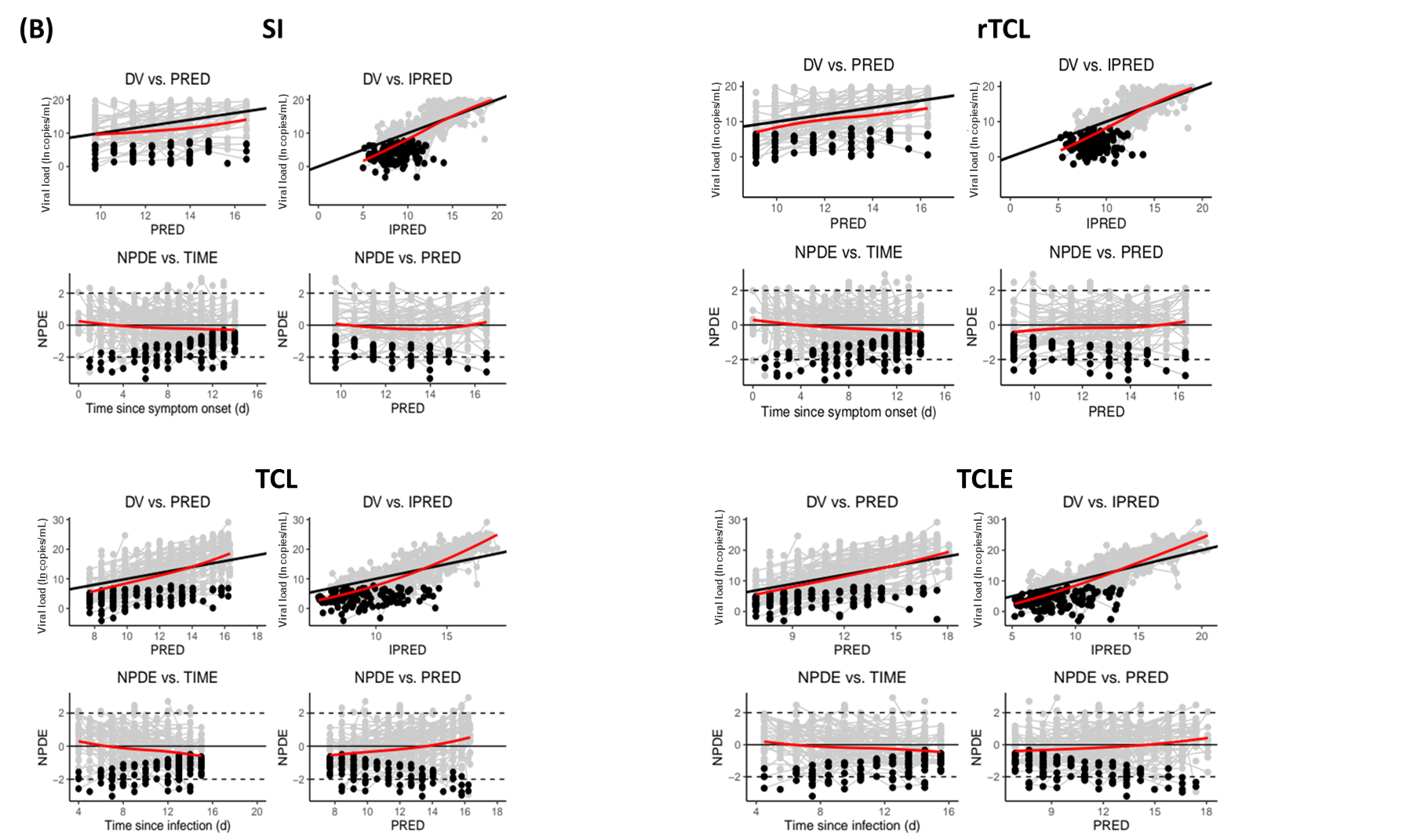


**Figure S3:** Goodness-of-fit plots for non-linear mixed effect model fitted to viral load. Grey dots are observed viral loads. Black dots are observed viral loads below the limit of detection (LOD). The black line indicates the line of identity. The red line represents the smooth curve. Panel (A) represents data extracted from Gastine et al (dataset A). Panel (B) represents data extracted from Néant et al (dataset B). SI: slope-intercept exponential decay model; rTCL: reduced target cell limited model; TCL: target cell limited model; TCLE: TCL with eclipse phase model. IPRED: individual predicted viral load; PRED: population predicted viral load; NPDE: normalised prediction distribution errors; DV: observed viral loads; ln: natural logarithm.


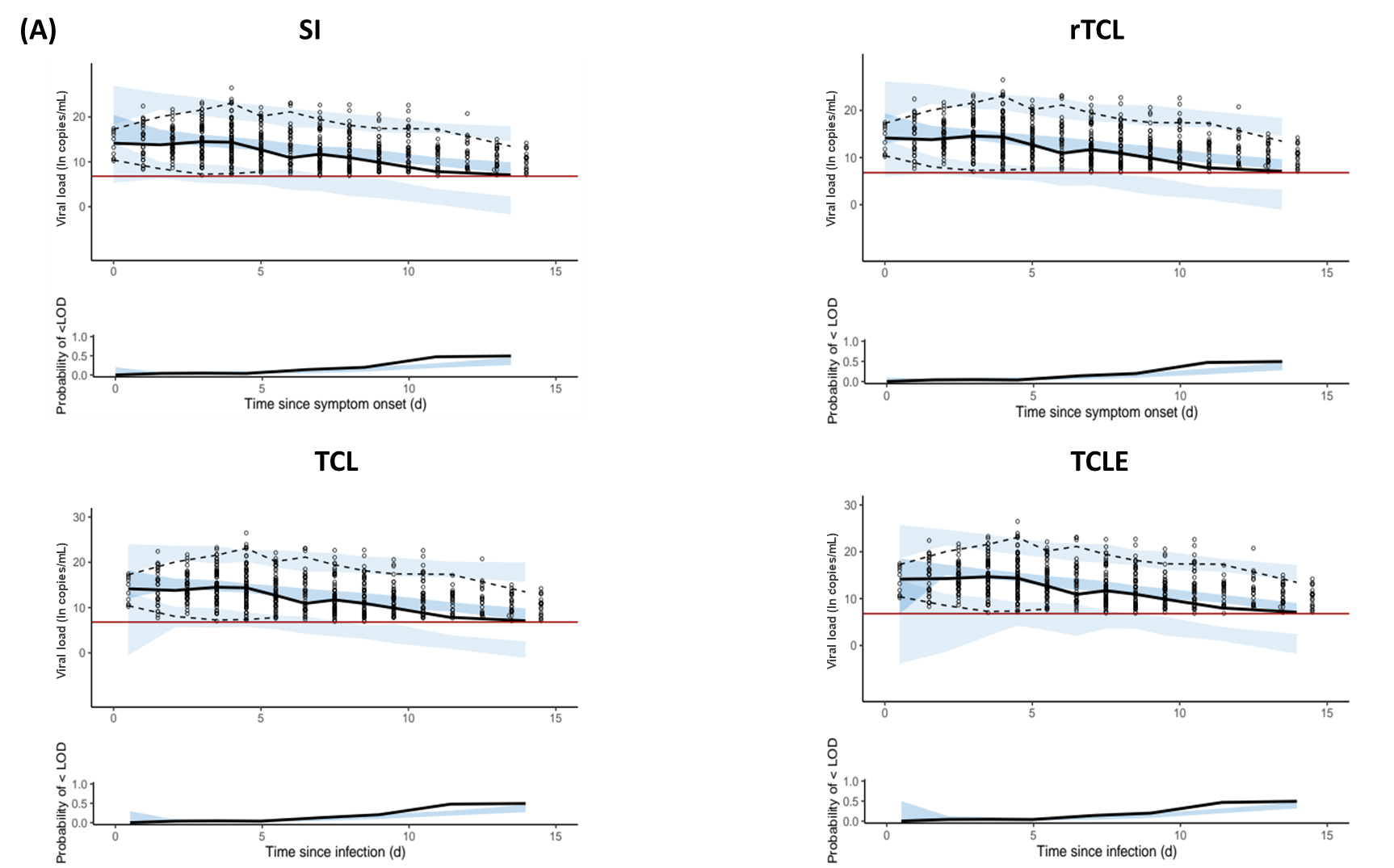


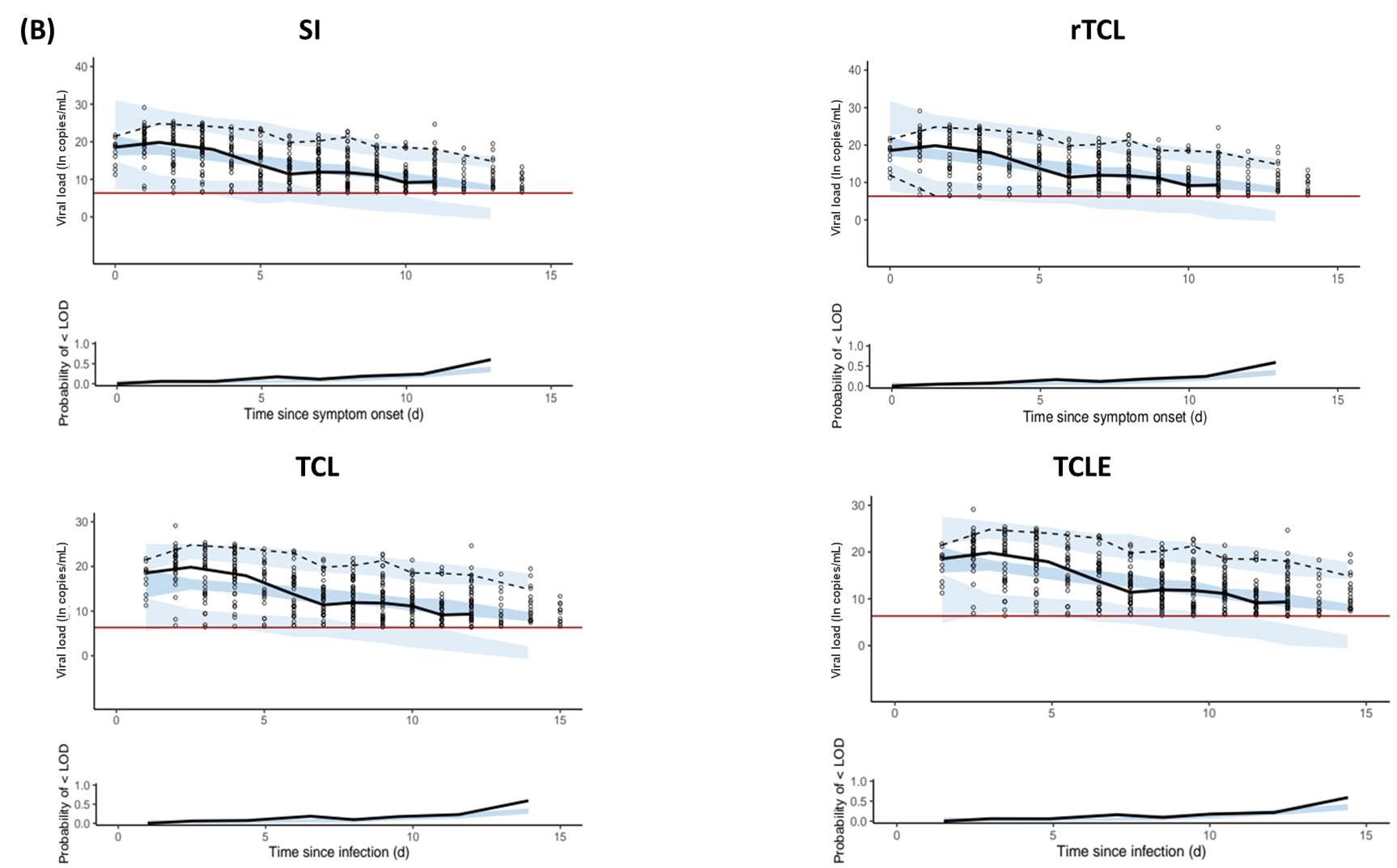


**Figure S4:** Visual predictive checks for non-linear mixed effect model fitted to viral load. Circles are observed viral loads. The red line indicates the limit of detection (LOD). In the top plot, the black line is the 50^th^ percentile of the observed data. The broken black lines represent the 95^th^ and 5^th^ percentiles of the observed data. The shaded areas represent the 90% prediction interval of the 95^th^, 50^th^ and 5^th^ percentiles of the simulated data. In the bottom plot, the black line represents the observed proportion of viral loads below the LOD, and the shaded area represents the 90% prediction interval of the simulated proportion of viral loads below the LOD. Panel (A) represents data extracted from Gastine et al (dataset A). Panel (B) represents data extracted from Néant et al (dataset B). SI: slope-intercept exponential decay model; rTCL: reduced target cell limited model; TCL: target cell limited model; TCLE: TCL with eclipse phase model; ln: natural logarithm.

**References**

1. Kim KS, Ejima K, Iwanami S, Fujita Y, Ohashi H, Koizumi Y, et al. A quantitative model used to compare within-host SARS-CoV-2, MERS-CoV, and SARS-CoV dynamics provides insights into the pathogenesis and treatment of SARS-CoV-2. PLoS Biol. 2021;19(3):e3001128.
2. Kamal MA, Gieschke R, Lemenuel-Diot A, Beauchemin CA, Smith PF, Rayner CR. A drug-disease model describing the effect of oseltamivir neuraminidase inhibition on influenza virus progression. Antimicrob Agents Chemother. 2015;59(9):5388-95.
3. Néant N, Lingas G, Le Hingrat Q, Ghosn J, Engelmann I, Lepiller Q, et al. Modeling SARS-CoV-2 viral kinetics and association with mortality in hospitalized patients from the French COVID cohort. Proc Natl Acad Sci U S A. 2021;118(8).
4. Dodds MG, Krishna R, Goncalves A, Rayner CR. Model-informed drug repurposing: Viral kinetic modelling to prioritize rational drug combinations for COVID-19. Br J Clin Pharmacol. 2021;87(9):3439-50.
5. Gastine S, Pang J, Boshier FAT, Carter SJ, Lonsdale DO, Cortina-Borja M, et al. Systematic Review and Patient-Level Meta-Analysis of SARS-CoV-2 Viral Dynamics to Model Response to Antiviral Therapies. Clin Pharmacol Ther. 2021;110(2):321-33.
